# Smoking prevalence differs by location of residence among Ghanaians in Africa and Europe: The RODAM study

**DOI:** 10.1371/journal.pone.0177291

**Published:** 2017-05-05

**Authors:** Rachel Brathwaite, Juliet Addo, Anton E. Kunst, Charles Agyemang, Ellis Owusu-Dabo, Ama de-Graft Aikins, Erik Beune, Karlijn Meeks, Kerstin Klipstein-Grobusch, Silver Bahendeka, Frank P. Mockenhaupt, Stephen Amoah, Cecilia Galbete, Matthias B. Schulze, Ina Danquah, Liam Smeeth

**Affiliations:** 1 Department of Non-communicable Disease Epidemiology, London School of Hygiene and Tropical Medicine, London, United Kingdom; 2 Department of Public Health, Academic Medical Center-University of Amsterdam, Amsterdam, The Netherlands; 3 Kumasi Centre for Collaborative Research in Tropical Medicine, Kwame Nkrumah University of Science and Technology, Kumasi, Ghana; 4 Department of Global Health, School of Public Health, KNUST, Kumasi, Ghana; 5 Regional Institute for Population Studies, University of Ghana, Legon, Ghana; 6 Julius Global Health, Julius Center for Health Sciences and Primary Care, University Medical Center Utrecht, The Netherlands; 7 Division of Epidemiology and Biostatistics, School of Public Health, Faculty of Health Sciences, University of the Witwatersrand, Johannesburg, South Africa; 8 International Diabetes Federation, Africa Region, Kampala, Uganda; 9 Institute of Tropical Medicine and International Health, Charité – Universitätsmedizin Berlin, Berlin, Germany; 10 Department of Molecular Epidemiology, German Institute of Human Nutrition Potsdam-Rehbruecke, Nuthetal, Germany; Harvard Medical School, UNITED STATES

## Abstract

**Background:**

Although the prevalence of smoking is low in Ghana, little is known about the effect of migration on smoking. Comparing Ghanaians living in their country of origin to those living in Europe offers an opportunity to investigate smoking by location of residence and the associations between smoking behaviours and migration-related factors.

**Methods:**

Data on a relatively homogenous group of Ghanaians living in London (n = 949), Amsterdam (n = 1400), Berlin (n = 543), rural Ghana (n = 973) and urban Ghana (n = 1400) from the cross-sectional RODAM (**R**esearch on **O**besity & **D**iabetes in **A**frican **M**igrants) study were used. Age-standardized prevalence rates of smoking by location of residence and factors associated with smoking among Ghanaian men were estimated using prevalence ratios (PR: 95% CIs).

**Results:**

Current smoking was non-existent among women in rural and urban Ghana and London but was 3.2% and 3.3% in women in Amsterdam and Berlin, respectively. Smoking prevalence was higher in men in Europe (7.8%) than in both rural and urban Ghana (4.8%): PR 1.91: 95% CI 1.27, 2.88, adjusted for age, marital status, education and employment.

Factors associated with a higher prevalence of smoking among Ghanaian men included European residence, being divorced or widowed, living alone, Islam religion, infrequent attendance at religious services, assimilation (cultural orientation), and low education.

**Conclusion:**

Ghanaians living in Europe are more likely to smoke than their counterparts in Ghana, suggesting convergence to European populations, although prevalence rates are still far below those in the host populations.

## Introduction

Worldwide, tobacco smoking is one of the strongest modifiable risk factors for chronic diseases.[1] Smoking prevalence differs across and within geographical world regions,[2] including sub-Saharan Africa (SSA).[3] Smoking prevalence in Ghana is considerably low, compared to other SSA and high income countries.[4, 5] Differences in smoking prevalence between migrants from the same country living in different locations were previously observed.[6] Smoking behaviour in migrant populations may change partly due to adopting the smoking norms of host populations,[7] and the influence of tobacco control policies, anti-smoking interventions,[8] socio-demographic characteristics,[9] religious affiliations,[10] family,[11] and community-level attitudes.[12]

Migrants from 3 SSA countries living in the US had significantly lower prevalences of smoking compared to non-migrant peers in their countries of origins.[13] Smoking data are unavailable for SSA migrants in Europe compared to SSA. Research conducted in England and Wales reported the prevalence of smoking was 4.4% among SSA migrant women,[14] which was higher than that seen among women in most African countries in SSA.[3] SSA men had a 14.4% prevalence of smoking in UK and Wales.[14] This was similar to the smoking prevalences observed among men in several SSA countries in recent studies. The smoking prevalence is much higher in the European region (approximately 35%) than the Americas (approximately 25%).[15] The factors which influence smoking behaviour among SSA migrants in Europe compared to the home countries are unknown. Preventing smoking uptake is a crucial step in reducing disproportionately increased burdens of cardiovascular diseases among African ethnic groups in Europe.[16]

This research aimed to describe smoking patterns in Ghanaians living in rural and urban Ghana compared to European cities, namely London, Amsterdam and Berlin and to determine the factors associated with smoking.

## Methods

### Study design and setting

Briefly, the RODAM Study (**R**esearch on **O**besity & **D**iabetes among **A**frican **M**igrants) is a multi-centre cross-sectional study of the prevalence and associated factors of obesity and diabetes among Ghanaians aged 25–70 living in London, Amsterdam, Berlin, rural and urban Ghana.[17]

Data were collected between 2012 and 2015 through structured questionnaires on socio-demographic factors, lifestyle practices, and health outcomes administered by trained research assistants. Ethical approval was granted by the relevant ethics committees in (School of Medical Sciences/Komfo Anokye Teaching Hospital Committee on Human Research, Publication & Ethical Review Board), the Netherlands (Institutional Review Board of the AMC, University of Amsterdam), Germany (Ethics Committee of Charite-Universitatsmedzin Berlin) and the UK (London School of Hygiene and Tropical Medicine Research Ethics Committee) prior to data collection.[17] Written informed consent was obtained from all participants.

### Study population

A Ghanaian was defined as either born in Ghana (first-generation) with at least one Ghana-born parent, or born elsewhere but both parents born in Ghana (second-generation). A multi-stage random sampling method was employed in Ghana using the list of enumeration areas in the Ashanti region stratified by urban and rural areas. In Amsterdam, Ghanaians were randomly selected from the Amsterdam Municipal Health register. In London, recruitment occurred through Ghanaian-based organisations and churches since no list of Ghanaian residents was available. In Berlin, a list of Ghanaian participants was provided by the registration office but due to low response to the written invitation, recruitment was changed to include Ghanaian-based organisations and churches as the sampling frame. From those invited, 76% in rural Ghana, 74% in urban Ghana, 75% in London and 68% in Berlin participated. In Amsterdam, 67% of those invited responded and of this 53% participated in the study.

### Smoking assessment

Determination of current smoker, ex-smoker or never smoker was based on either a ‘Yes’, ‘No, but I used to smoke’ or ‘No, I’ve never smoked’ response to the question ‘Do you smoke at all?’.

### Assessment of covariates

Questionnaire items included, among others, marital status, household composition, religious practises, frequency of engagement with religious activities, educational level, employment status (employed vs unemployed), occupational class (manual or non-manual), duration of residence in Europe and age at migration to Europe.

Berry’s model of acculturation was assessed using the bi-dimensional perspective; cultural orientation and ethnic identity (psychological domains), and social networks (behavioural domain).[18] This conceptualised the degree of retention or attachment of participants to both the original Ghanaian culture and the Dutch/German/English culture. Cultural orientation was measured using the Psychological Acculturation Scale.[19] Social networks was determined from the number of and time spent with Dutch/German/English friends. Ethnic identity was determined from the degree to which individuals felt Ghanaian/Dutch/German/English. Scores were assigned using a 5 point Likert scale. Mean scores were then grouped into Yes/No if ≥3 or <3 respectively. Acculturation levels were categorized into four: 1) Integration: adaptation to the host culture without losing attachment to the original culture. 2) Assimilation: cultural adaptation to the host culture accompanied by loss of original culture. 3) Separation: rejection to host culture and orientation to original culture. 4) Marginalization: rejection of both host and culture of origin.

### Data analysis

Using Stata version 14.1, general characteristics, using numbers and percentages, stratified by location of residence and gender were reported. Between group comparisons utilised chi-squared tests for categorical variables, mean difference and t-tests for continuous normally distributed variables and median (95% CIs) for non-normally distributed continuous variables. To uphold the RODAM participant inclusion criteria only the 25 to 70 age group was selected.[17] Age-standardised prevalence rates of current and ex-smokers were determined using the entire RODAM population as the standard population stratified by 10-year age groups.

Due to the few male smokers in Ghana (n = 35) and Europe (n = 94), the factors associated with smoking were evaluated in the total male population, first adjusting for age only, then for age, marital status, education and employment simultaneously to control for possible confounding.

In order to determine whether the effect of residence (Ghana vs Europe) on smoking varied by gender, interactions were tested for using likelihood ratio tests. These were also performed to assess potential interactions with other factors (religion, education and marital status) associated with smoking from the overall analysis among men.

Regression analysis of associations between migration-related factors and smoking were restricted to male first generation migrants. Due to the few female Ghanaian smokers (n = 28), regression analysis for factors associated with current smoking was not performed.

## Results

### Study population

**Fig 1:**
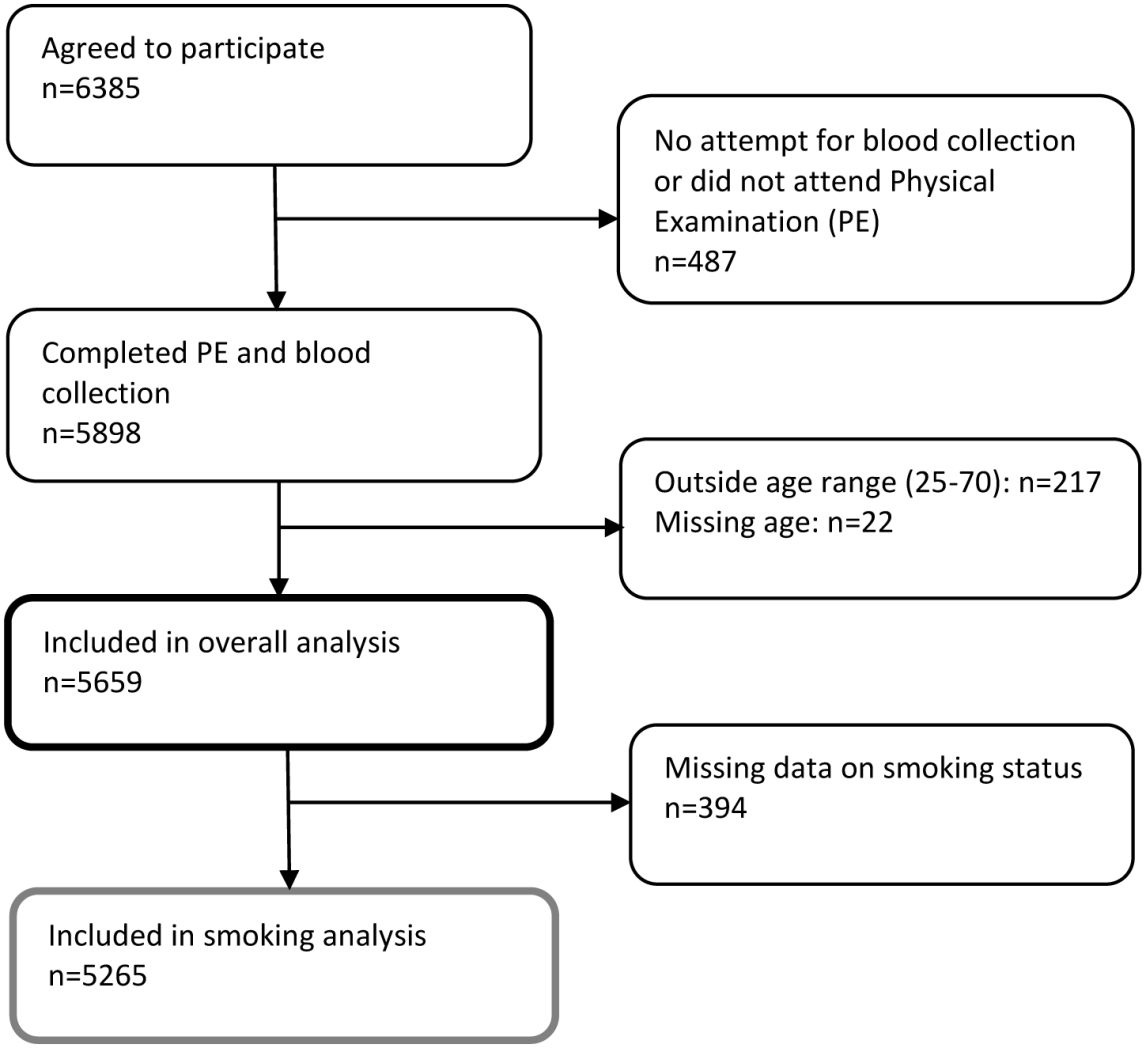
Flow chart showing inclusion of RODAM participants in smoking analysis.

In total 6,385 Ghanaians agreed to participate across all study locations. From 5,659 participants who attended the physical examination, 5,265 with data on smoking were included in this analysis (Fig 1).

There were no statistically significant differences in the age, gender, marital status and educational level of persons who did and who did not provide information on smoking status (n = 394). The sample comprised 62.3% of women and 37.7% of men. Men (mean age, 46.9 ± SD 11.1) were slightly older than women (45.8 ± SD 10.7), p<0.001. Most participants were married except in Amsterdam (Table 1). In London, most participants were highly educated and worked in non-manual full-time occupations. The majority practiced Christianity. Most first generation migrants settled in the European cities between 25 and 35 years (50.6% of men and 44.5% of women), and the median duration of residence in Europe was 18 years.

**Table 1:** Socio-cultural and migration-related characteristics of male and female Ghanaians in each study location.

Characteristics	Rural Ghana N = 973		Urban Ghana N = 1400		London N = 949		Amsterdam N = 1400		Berlin N = 543	
	Men	Women	Men	Women	Men	Women	Men	Women	Men	Women
**Number of participants, N**	**381**	**592**	**392**	**1008**	**362**	**587**	**553**	**847**	**297**	**246**
**Age, Mean (SD)**	46.2 (12.8)	46.5 (12.5)	46.6 (11.8)	44.7 (11.2)	46.5 (10.8)	47.7 (10.5)	48.6 (9.3)	45.5 (8.7)	45.8 (11.1)	44.6 (9.3)
**Marital status, n (%)**										
Married or registered partnership	250 (65.6)	300 (50.7)	284 (72.5)	527 (52.3)	281 (77.6)	335 (57.1)	137 (24.8)	134 (15.8)	143 (48.2)	117 (47.6)
Cohabitating (living together)	66 (17.3)	91 (15.4)	24 (6.1)	91 (9.0)	6 (1.7)	15 (2.6)	133 (24.1)	157 (18.5)	31 (10.4)	15 (6.1)
Unmarried (never married)	27 (7.1)	21 (3.6)	59 (15.1)	77 (7.6)	35 (9.7)	62 (10.6)	154 /(27.9)	243 (28.7)	77 (25.9)	39 (15.9)
Divorced or separated	32 (8.4)	84 (14.2)	21 (5.4)	171 (17.0)	15 (4.1)	118 (20.1)	119 (21.5)	286 (33.8)	44 (14.8)	68 (27.6)
Widow/widower	5 (1.3)	94 (15.9)	3 (0.8)	136 (13.5)	3 (0.8)	29 (4.9)	3 (0.5)	11 (1.3)	0 (0.0)	6 (2.4)
**Highest level of education, n (%)**										
Never been to school or elementary schooling only	158 (41.5)	396 (66.9)	92 (23.5)	522 (51.8)	16 (4.4)	66 (11.2)	122 (22.1)	359 (42.4)	18 (6.1)	29 (11.8)
Lower vocational schooling or lower secondary schooling	146 (38.2)	164 (27.7)	176 (44.9)	371 (36.8)	102 (28.2)	198 (33.7)	235 (42.5)	277 (32.7)	142 (47.8)	135 (54.9)
Intermediate vocational schooling or intermediate higher secondary schooling	54 (14.2)	19 (3.2)	85 (21.7)	88 (8.7)	67 (18.5)	162 (27.6)	143 (25.9)	161 (19.0)	84 (28.3)	62 (25.2)
Higher vocational schooling or university	23 (6.0)	12 (2.0)	38 (9.7)	27 (2.7)	168 (46.4)	148 (24.2)	49 (8.9)	33 (3.9)	52 (17.5)	19 (7.7)
**Religion, n (%)**										
Christian-based religion	197 (51.7)	365 (61.7)	234 (59.7)	635 (63.0)	295 (81.5)	517 (88.1)	398 (72.0)	653 (77.1)	185 (62.3)	217 (88.2)
Islamic	35 (9.2)	23 (3.9)	41 (10.5)	99 (9.8)	0 (0.0)	0 (0.0)	16 (2.9)	16 (1.9)	18 (6.1)	2 (0.8)
Other religion	22 (5.8)	39 (6.6)	6 (1.5)	28 (2.8)	14 (3.9)	13 (2.2)	31 (5.6)	53 (6.3)	5 (1.7)	4 (1.6)
**Frequency of attending religious services, n (%)**										
Once a week	226 (59.3)	388 (65.5)	260 (66.3)	731 (72.5)	280 (77.4)	492 (83.8)	304 (55.0)	571 (67.4)	304 (55.0)	175 (71.1)
Less than once a week	24 (6.3)	34 (5.7)	15 (3.8)	32 (3.2)	28 (7.7)	27 (4.6)	102 (18.4)	112 (13.2)	102 (18.4)	41 (16.7)
Never	2 (0.5)	3 (0.5)	6 (1.5)	1 (0.1)	0 (0.0)	2 (0.3)	27 (4.9)	21 (2.5)	27 (4.9)	5 (2.0)
**Employment status, n (%)**										
Employed	340 (89.2)	535 (90.4)	336 (85.7)	856 (84.9)	305 (84.3)	437 (74.5)	390 (70.5)	450 (53.1)	213 (71.7)	151 (61.4)
Unemployed	40 (10.5)	56 (9.5)	56 (14.3)	152 (15.1)	48 (13.3)	135 (23.0)	160 (28.9)	373 (44.0)	82 (27.6)	94 (38.2)
**Occupational class, n (%)**										
Non-manual	46 (12.1)	69 (11.7)	122 (31.1)	364 (36.1)	202 (55.8)	328 (55.9)	84 (15.2)	100 (11.8)	78 (26.3)	78 (31.7)
Manual	320 (84.0)	486 (82.1)	260 (66.30	569 (56.5)	120 (33.2)	201 (34.2)	316 (57.1)	373 (44.0)	182 (61.3)	135 (54.9)
**Immigrant generation, n (%)**										
First	n/a	n/a	n/a	n/a	350 (96.7)	563 (95.9)	544 (98.4)	841 (99.3)	295 (99.3)	245 (99.6)
Second	n/a	n/a	n/a	n/a	12 (3.3)	21 (3.6)	8 (1.5)	6 (0.7)	2 (0.7)	1 (0.4)
**Duration of years of residence in European area among first generation migrants, Median (95% CI)**	n/a	n/a	n/a	n/a	13 (13, 14)	15 (14, 16)	22 (20, 23)	19 (19, 20)	18 (16, 20)	18 (16, 21)
**Age at first migration, Median (95% CI) years**	n/a	n/a	n/a	n/a	29 (28, 31)	28 (27, 30)	29 (29, 30)	27 (26, 28)	28 (27, 29)	26 (25, 27)
**Age group at first migration among first generation migrants, n (%)**	n/a	n/a	n/a	n/a						
>25 years	n/a	n/a	n/a	n/a	247 (70.6)	346 (61.5)	413 (75.9)	551 (65.5)	213 (72.2)	151 (61.6)
16–25 years	n/a	n/a	n/a	n/a	68 (19.4)	156 (27.7)	59 (16.4)	185 (22.0)	65 (22.0)	77 (31.4)
Less than 16 years	n/a	n/a	n/a	n/a	13 (3.7)	23 (4.1)	33 (6.1)	82 (9.8)	12 (4.1)	12 (4.9)

n/a- not applicable

### Smoking prevalence

**Fig 2:**
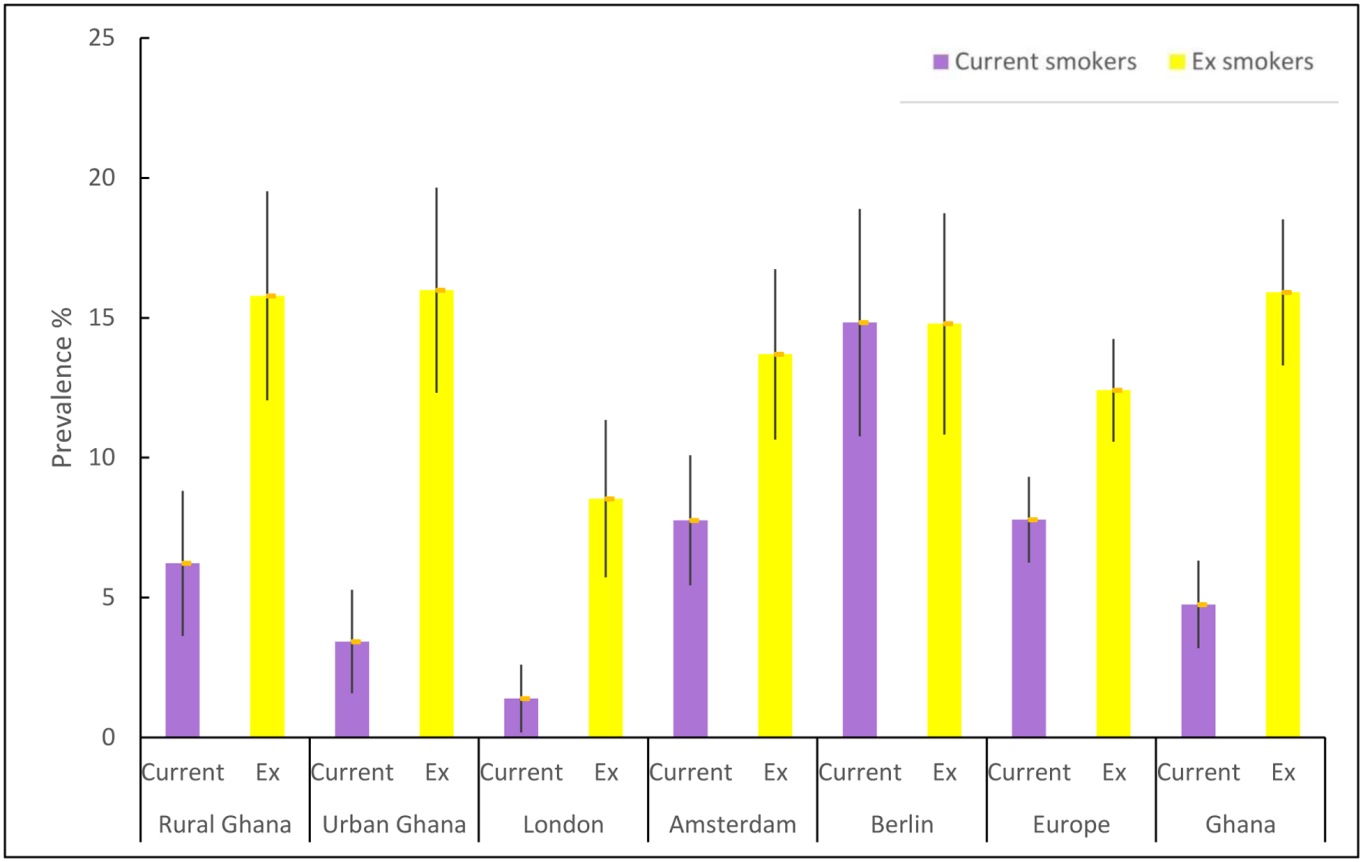
Age-standardized prevalence of current and ex-smokers among men.

**Fig 3:**
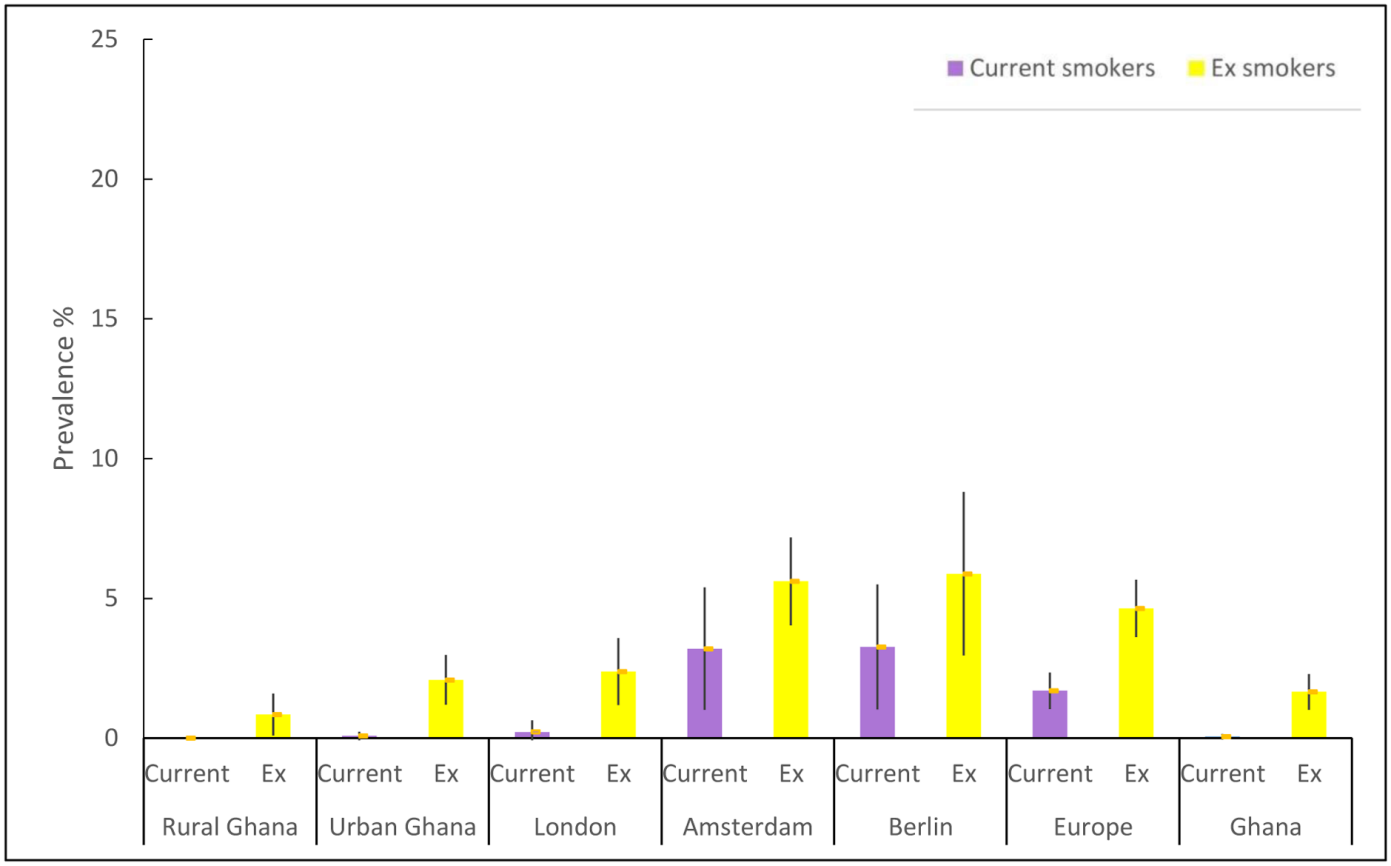
Age-standardized prevalence of current and ex-smokers among women.

The prevalence of smoking was lower in Ghana (1.5%) than Europe (4.2%), p<0.001, in men, 4.8% and 7.8% respectively, p<0.01. Fig 2 shows the age-standardized prevalence rates of current and ex-smokers among Ghanaians in each location of residence for men, and Fig 3 for women.

Current smoking was most common among Ghanaian men in Berlin (14.8%; 95% CI 10.8% to 18.9%) followed by Amsterdam (7.8%; 95% CI 5.4% to 10.0%), rural Ghana (6.2%; 95% CI 3.6% to 8.6%) and urban Ghana (3.4%; 95% CI 1.6% to 5.3%). Smoking was much less common in men in London (1.4%, 95% CI 0.2% to 2.6%).

For women, current smoking was, again, most common in Berlin (3.3%; 95% CI 1.0% to 5.5%), followed by Amsterdam (3.2%; 95% CI 1.0% to 5.4%), London (0.2%, 95% CI -0.2% to 0.6%), and urban Ghana (0.1%, 95% CI -0.1% to 0.2%) while no women smoked in rural Ghana.

#### Patterns of smoking and ex-smoking

Current smokers smoked a median of 5 (95% CI, 4–6) cigarettes per day. The median length of time ex-smokers smoked before quitting was 7 years (95% CI, 5–10) for men and 3 years (95% CI, 2–6) for women. Most ex-smokers quitted smoking more than a decade ago (66.2%). Approximately 6% of all ex-smokers recently quitted smoking (less than 1 year previously).

### Factors associated with smoking among men

After adjusting for age, marital status, education and employment, residence, marital status, living arrangements, religion practiced, frequency of attending religious services and educational level were associated with current smoking (Table 2). Within Ghana, there was no difference in smoking prevalence by location.

**Table 2:** Factors associated with current smoking among Ghanaian men in all locations.

	Total men	Current smokers		
Characteristics		n (%)	Adjusted for age only PR (95% CI)	Adjusted for age, marital status, education and employment PR (95% CI)
**All men**	**N = 1985**			
**Age group**				
25–34	368	20 (5.4)	1.00*	1.00
35–44	428	27 (6.3)	1.16 (0.66, 2.03)	1.15 (0.64, 2.07)
45–54	638	46 (7.2)	1.33 (0.80, 2.21)	1.23 (0.71, 2.13)
55–70	551	36 (6.5)	1.20 (0.71, 2.04)	1.08 (0.60, 1.93)
**Site**				
Rural Ghana	381	22 (5.8)	1.00	1.00
Urban Ghana	392	13 (3.3)	0.57 (0.29, 1.12)	0.64 (0.33, 1.27)
Both urban and rural Ghana	773	35 (4.5)	1.00	1.00
London	362	5 (1.4)	**0.30 (0.12, 0.77)**	0.56 (0.21, 1.49)
Amsterdam	553	45 (8.1)	**1.75 (1.14, 2.70)**	**1.74 (1.02, 2.96)**
Berlin	297	44 (8.1)	**3.26 (2.13, 4.99)**	**3.81 (2.35, 6.16)**
All European locations	1212	94 (7.8)	**1.69 (1.15, 2.47)**	**1.91 (1.27, 2.88)**
**Marital status**				
Married or cohabitating	1095	57 (5.2)	1.00	1.00
Never married	260	16 (6.2)	1.22 (0.71, 2.08)	0.99 (0.57, 1.74)
Divorced or widowed	597	56 (9.4)	**1.93 (1.32, 2.82)**	**1.71 (1.15, 2.53)**
**Household**				
Living with children	417	16 (3.8)	1.00	1.00
Living alone	157	23 (14.7)	**3.98 (2.08, 7.59)**	**2.88 (1.51, 5.48)**
Living with family or other adults	266	23 (5.7)	**2.36 (1.24, 4.49)**	1.86 (0.99, 3.51)
**Religion**				
Christian-based religion	1309	53 (4.1)	1.00	1.00
Islam religion	110	12 (10.9)	**2.73 (1.51, 4.94)**	**2.43 (1.30, 4.54)**
Other religions or faiths	78	5 (6.4)	1.57 (0.65, 3.82)	1.60 (0.65, 3.89)
Frequency of attending religious services*				
Once a week or more	1200	45 (3.8)	1.00	1.00
Less than once a week	236	20 (8.5)	**2.28 (1.37, 3.79)**	**1.98 (1.16, 3.36)**
Never	46	4 (8.7)	2.27 (0.84, 6.15)	2.28 (0.82, 6.33)
**Education**				
Lower vocational and below	1207	96 (8.0)	1.00	1.00
Intermediate vocational/ higher secondary	433	28 (6.5)	0.82 (0.54, 1.24)	0.85 (0.56, 1.28)
Higher vocational school/ university	330	5 (1.5)	**0.19 (0.08, 0.48)**	**0.23 (0.09, 0.58)**
**Employment**				
Employed	1584	94 (5.9)	1.00	1.00
Unemployed	386	33 (8.6)	1.48 (1.00, 2.18)	1.33 (0.89, 1.98)
**Occupational class**				
Non-manual	532	25 (4.7)	1.00	1.00
Manual	1198	78 (6.5)	1.36 (0.88, 2.12)	0.91 (0.58, 1.41)
**First generation men**^α^	**N = 1678**^α^			
**Duration of residence in European area (years)**	n/a	n/a	1.01 (0.98, 1.03)	1.10 (1.00, 1.21)
**Duration of residence in European area (categories)**				
Less than 10 years	224	20 (8.9)	1.00	1.00
10 to 19 years	285	17 (6.0)	0.90 (0.40, 2.06)	0.93 (0.41, 2.09)
20 to 29 years	302	24 (8.0)	1.08 (0.47, 2.47)	0.92 (0.39, 2.16)
30 or more years	62	10 (16.1)	2.10 (0.76, 5.80)	1.68 (0.58, 4.87)
**Age at first migration (years)**	n/a	n/a	0.98 (0.65, 1.02)	0.99 (0.95, 1.03)
**Acculturation: Cultural Orientation**				
Integration	685	54 (7.9)	1.00	1.00
Assimilation	6	3 (50.0)	**6.77 (3.25, 14.11)**	**9.51 (3.36, 26.92)**
Separation	214	15 (7.0)	0.90 (0.52, 1.56)	0.61 (0.35, 1.09)
Marginalization	4	1 (25.0)	3.07 (0.60, 15.78)	1.09 (0.28, 4.29)
**Acculturation: Ethnic identity**				
Integration	481	41 (8.5)	1.00	1.00
Assimilation	19	0 (0.0)	-	-
Separation	371	30 (8.1)	0.94 (0.60, 1.48)	1.02 (0.65, 1.61)
Marginalization	38	2 (5.3)	0.70 (0.17, 2.82)	1.28 (0.32, 5.15)
**Acculturation: Social Network**				
Integration	512	37 (7.2)	1.00	1.00
Assimilation	147	12 (8.2)	1.07 (0.57, 2.01)	1.12 (0.59, 2.14)
Separation	155	15 (9.7)	1.33 (0.75, 2.36)	1.23 (0.69, 2.19)
Marginalization	95	9 (9.5)	1.31 (0.66, 2.62)	1.25 (0.62, 2.52)

*Only age in the model;

n/a-not applicable;

^**α**^ first generation migrants who migrated after age 25 years

Men in Europe were 1.9 times more likely to smoke than men in Ghana but smoking prevalence in Ghana and London was similar. In contrast, men in Amsterdam and Berlin were 1.7 times and 3.8 times, respectively, more likely to smoke than men in Ghana.

Divorced or widowed men were 1.7 times more likely to smoke than married or cohabitating men (Table 2). Men living alone were 2.9 times more likely to smoke than men who lived with children. Further positively associated factors included Islam religion, rare attendance at religious services, and lower secondary or no education.

#### Interactions with location

The effect of location (Ghana vs Europe) on smoking differed significantly with gender (p<0.001). Ghanaian women in Europe were 12 times more likely to smoke than women in Ghana (PR 12.42; 95% CI, 1.34, 115.35), while this difference was only 2-fold for men (PR 1.99; 95% CI, 1.26–3.14) after adjusting for age, marital status, education, and employment.

No other significant interactions between location and religion, education and marital status were present.

### Migration-related factors and smoking

Among first generation men who migrated to Europe at the age of 25 or older, duration of residence and age of migration were not significantly associated with smoking (Table 2). Men whose cultural orientation showed signs of assimilation were more likely to smoke than integrated men. Ethnic identity and social networks were not associated with smoking.

#### Smoking initiation and migration

Among the 88 current smokers who were first generation migrants and migrated at age 25 or older, the majority started smoking while living in Europe (Table 3). 57.1% of men started smoking in Amsterdam but in Berlin, 51.4% had started smoking before migrating. Among women, 53.6% of women started smoking in Amsterdam. No one smoked before going to London. Of the women who were smokers in Berlin, 2 started smoking in Ghana and the other 2 in Berlin respectively.

**Table 3:** Period when first generation current smokers started smoking by gender and European location of residence (restricted to those who migrated after age 25).

Gender	Period when started smoking	Amsterdam n (%)	Berlin n (%)	London n (%)	All European Locations n (%)
Men	Total current smokers	35	35	3	73
	Before migration*	15 (42.9)	18 (51.4)	0 (0.0)	33 (45.2)
	After migration**	20 (57.1)	17 (48.6)	3 (100.0)	40 (54.8)
Women	Total current smokers	11	4	0 (0.0)	15
	Before migration*	4 (36.4)	2 (50.0)	0 (0.0)	6 (40.0)
	After migration**	7 (63.6)	2 (50.0)	0 (0.0)	9 (60.0)

*assuming when in Ghana;

**while residing in European location

## Discussion

### Key findings

Smoking is remarkably uncommon among Ghanaians when compared to European populations, and particularly so for Ghanaians in their home country. Among women, smoking in Ghana and London is almost non-existent and still rare in Amsterdam and Berlin. Among men, the prevalence of smoking is higher in Amsterdam and Berlin (but not in London) than in Ghana but, overall, far below the European average. More than half of first generation men and the majority of women started smoking in Europe. All smokers were light smokers.

Among Ghanaian men in Europe, migration-related factors including duration of residence and age at migration were not associated with smoking. Assimilation in the context of cultural orientation was associated with an increase in smoking, but not in other measures including ethnic identity or social networks.

### Discussion of key findings

The prevalence of smoking among migrant Ghanaian populations showed patterns of convergence to the European population since it reflected the ranking of smoking among adults in the respective locations. Smoking prevalence was highest in Berlin, followed by Amsterdam and London. Recent national estimates indicated smoking prevalence in men and women of 33% and 27% in Germany,[20] of 29% and 23% in the Netherlands,[21] and of each 22% in the UK.[21] The Office for National Statistics 2015 data for the UK reported 19.3% of men and 15.3% of women smoked cigarettes.[22] The finding that smoking was substantially lower among men in London compared to Amsterdam and Germany is striking, since a prevalence of 14.3% for current cigarette smoking was observed for SSA migrant men in the UK and Wales in general.[14] This discrepancy supports the importance of collecting data on country of birth/ethnicity in population surveys and routine data,[23] since the heterogeneity of the population contributes to loss of details which are useful for preparing targeted interventions.[24]

The low prevalence of smoking among Ghanaian men in Ghana corresponded to data in neighbouring West African countries including Nigeria,[25] and Benin,[26] as well as in Ethiopia and Sao Tome & Principe.[26] These similarities may relate to the substantial Christian population.[27] In other SSA countries, the smoking prevalence in men is much higher,[26] possibly due to cultural attitudes and ethnic compositions. The very low smoking prevalence among women in Ghana accords with previous studies,[5] and in most other SSA countries suggesting a widespread cultural norm discouraging women from smoking.[3]

Most first generation migrant smokers started smoking only in Europe alluding to respective influences of the European environment with its substantially greater proportion of smokers in native European populations. Smokers usually begin smoking before 18 years.[28] Interestingly, the average age of migration among Ghanaian men who smoked in Europe was 29 years (95% CI: 27 to 30). This indicated that smoking began on average much later among the 54.8% of male smokers in Europe, as compared to European-origin populations.[29] The smoking initiation at a later age among Ghanaian migrants in Europe is consistent with the patterns of smoking in Ghana, where smoking was more common among older age groups.[5]

More non-smokers migrated to London as compared to Amsterdam and Berlin, suggesting selective movement of Ghanaian smokers and non-smokers between the locations.[30] It might be that the non-smokers may have migrated to London for reasons such as education and employment opportunities as was supported by the higher proportion of highly educated participants in London than Berlin and Amsterdam (46%, 17.5% and 8.7% of men respectively). This suggests the non-smokers were probably more economically capable of migrating to London than smokers who may be of lower socio-economic status.

Within Europe, the link between assimilation and smoking was inconsistent as it was only present for cultural orientation and not for measures of ethnic identity and social behaviour. The difference in smoking prevalence between Ghanaian women in Ghana and Europe hinted to the influence of Western lifestyles (a form of unhealthy assimilation).[31] Other research showed that strong ethnic pride and identity can be protective against unhealthy behaviours including smoking.[32] The inconsistency of our findings regarding the impact of acculturation suggests that any form of convergence is halted in the Ghanaian migrant community but requires further elucidation. This implies that those protective factors comprising beliefs associated with Christianity, family values, and collectivism are still influential in the Ghanaian migrant community.

In the USA, assimilation created a negative effect on quitting for immigrants from countries with lower smoking rates than USA, while this positively influenced quitting for immigrants from countries with higher smoking rates than USA.[31] Although Ghana has lower smoking rate than Europe, we found no conclusive support assimilation’s effect on increased smoking in the Ghanaian community.

Higher smoking among Islamic Ghanaian men was consistent with the substantially increased prevalence of smoking in Islamic populations globally.[33] The once neutral position on smoking in the Islamic world may contribute to the higher rates still observed today.[33] Even though, respective discouragement is increasing also in the Islamic community.[34]

Being divorced or widowed accompanied increased prevalence of smoking compared to married men. Marriage or cohabitating has been linked to better health through its support (financial, psychological, social) for healthier behaviours.[35, 36] This falls in line with our finding that living with a child or children in the household prevents smoking.

Higher levels of education accompanied lower prevalence of smoking compared to men with lower levels of education. This supports presence of a socio-economic gradient for smoking,[37] even among immigrant males.[38] This pattern corresponds to the last stages of the smoking epidemic rather than the initial stages, where smoking is more common among higher educational groups. This observation suggested that the smoking epidemic had already peaked among Ghanaian men, despite their lower prevalence and consumption rates as compared to native European populations. Since the most highly educated male Ghanaians lived in London, this may explain part of the lower prevalence of smoking reported in London itself given the trends discussed previously. Possibly suggesting that a type of selective migration may have occurred among Ghanaian men with different kinds of people migrating to London than to Amsterdam and Berlin or for different reasons education being a major one of them.

### Strengths and limitations

This study’s strengths include the restriction to a relatively homogenous population of Ghanaians. In many studies on smoking, persons of African ancestry are often combined due to uncertainty on the actual origin. Using standardised questions across all sites removed the problem of variable smoking definitions. Standard population-based recruitment strategies were employed. Selection bias may result from the recruitment of many participants from churches in London and Berlin, and Christians may be over-represented. However, the majority of the population of Ghana practices Christianity (71.2%), with smaller proportions practicing Islam (17.6%), mostly in the North of the country) or traditional religions (5.2%).[39] Future work should explore potential options and their feasibilities in recruiting SSA migrant populations in European countries where population registers for these populations are lacking, for example name algorithms or more focused enumeration using ethnic group/country of birth at the Lower Layer Super Output Areas level.[40] Smoking may be under-reported in our study population due to a tendency towards responses considered socially desirable.[41, 42] The use of objective measures to confirm smoking status, such as urine analysis of nicotine metabolites, unfortunately was not conducted.[43] The small number of smokers impaired the preciseness of some of the estimates on smoking risk factors. However, the sample size was sufficient to detect a significant difference in smoking prevalence by location. Key parameters of respondents and non-respondents did not differ significantly.

### Implications

This study draws attention to environmental or societal influences on the smoking behaviour of ethnic minority groups from SSA in high-income European countries. Although smoking is still rare among Ghanaian women, their higher taking up of smoking as compared to males points to the vulnerability of female migrants and ethnic minority groups in high-income countries. Particular attention should be placed on migrants from countries with low smoking prevalence.

Higher rates of smoking upon migration may further increase the burden of tobacco-related diseases including cardiovascular disease and diabetes among ethnic minority migrant populations in Europe.[44, 45] Although this may not be realised among Ghanaian women since their smoking prevalence and intensity rates are substantially lower compared to native European women. Longitudinal studies following up migrant populations, which track the change in smoking status would be ideal for answering the question on the impact of migration on smoking behaviour.

## Conclusions

Smoking behaviour among this migrant population is multifactorial in nature, with exposure to a high smoking environment having a strong influence although rates are still far below the European populations. Research on the impact of location on women’s attitude towards smoking would help elucidate the societal factors underlying the lower smoking uptake in some locations and higher in others.

## Supporting information

S1 Checklist Research checklist. (DOC)
